# Effects of 12-week supplementation of marine Omega-3 PUFA-based formulation Omega3Q10 in older adults with prehypertension and/or elevated blood cholesterol

**DOI:** 10.1186/s12944-017-0617-0

**Published:** 2017-12-27

**Authors:** Tian Shen, Guoqiang Xing, Jingfen Zhu, Shuxian Zhang, Yong Cai, Donghua Li, Gang Xu, Evan Xing, Jianyu Rao, Rong Shi

**Affiliations:** 1 Department of Community Health and Behavior Medicine, School of Public Health, Shanghai Jiao Tong University, Shanghai, 200025 People’s Republic of China; 2 Imaging Institute of Rehabilitation and Development of Brain Function, the Second Clinical Medical College of North Sichuan Medical College, Nanchong Central Hospital, Nanchong, 637000 People’s Republic of China; 3 Lotus Biotech.com LLC, John Hopkins University-MCC, 9601 Medical Center Drive, Rockville, MD 20850 USA; 4 Tang Qiao Community Health Service Center, Pudong New District, Shanghai, 200127 People’s Republic of China; 5 Biochemistry Program, University of Maryland, Baltimore, MD 21201 USA; 6 Department of Pathology and Laboratory Medicine, David Geffen School of Medicine, University of California, Los Angeles, CA 90095 USA; 7 School of Public Health, Shanghai University of Traditional Chinese Medicine, Shanghai, 201203 People’s Republic of China; 8 Department of Community Health and Family Medicine, Shanghai Jiao Tong University School of Public Health, Shanghai, 200025 People’s Republic of China

**Keywords:** Fish oil-derived PUFA, Soybean oil, Blood pressures, Dyslipidemia, Old adults

## Abstract

**Backgrounds:**

To study the effects of supplementation of a marine omega-3 poly-unsaturated fatty acids (n3-PUFA) formulation (Omega3Q10) in older adults with hypertension and/or hypercholesterolemia.

**Methods:**

A total of 97 people were enrolled to receive 12-week supplementation of either Omega3Q10 (*n* = 48) or soybean oil (*n* = 49). Total cholesterol (TC), low density lipoprotein cholesterol (LDL-C), high density lipoprotein cholesterol (HDL-C), and hypertension-related symptoms were determined before and after the supplementation.

**Results:**

There were no baseline differences between the two groups. Omega3Q10 supplementation significantly reduced diastolic blood pressure (DBP) (from 81.6 ± 5.3 mmHg to 79.3 ± 5.2 mmHg, *P* < 0.05). Blood concentrations of TC and LDL-C decreased significantly and blood HDL-C level increased significantly after 12 weeks of Omega3Q10 (5.5 ± 0.7 vs. 5.3 ± 0.5, *P* < 0.05; 3.7 ± 0.8 vs. 3.3 ± 0.6, *P* < 0.05; 1.2 ± 0.6 vs. 1.3 ± 0.5, *P* < 0.05, respectively) and soybean oil supplementation (5.7 ± 0.8 vs. 5.6 ± 0.7, *P* < 0.05; 3.6 ± 0.7 vs. 3.4 ± 0.8, P < 0.05; 1.0 ± 0.8 vs. 1.2 ± 0.7, *P* < 0.05, respectively) but no group differences were found. A significantly greater proportion of the people in the Omega3Q10 group became free from headache and palpitations & chest tightness symptoms after the 12-week supplementation compared to that of the soybean oil group (95.5% vs. 71.4%, *P* < 0.01; 95.8 vs. 75.5%, *P* < 0.01, respectively).

**Conclusion:**

12-week supplementation of Fish oil-based PUFA appear to be more effective in improving DBP and hypertension-related symptoms than soybean oil in old adults with hypertension and hypercholesterolemia although both supplementation improved TC, LDL-C and HDL-C concentrations.

## Background

Elevated blood-pressure or blood cholesterol levels are major risk factors for cardiovascular events that are the major cause of death worldwide [1]. Hypertension that develops over time without a specific cause is considered benign hypertension. The common manifestations of hypertension include dizziness, headache, fatigue, tinnitus, palpitations & chest tightness, and lassitude in the loins and knees. It is estimated that more than one third of adults living in the US and China has hypertension or hypercholesterolemia, most of whom remain untreated [2, 3].

While modification of lifestyle, including low-sodium intake, high potassium diet, increasing physical activity, quitting smoking, reducing alcohol consumption, and maintaining a healthy weight can lower the risk of one’s progression of hypertension, many people experience difficulties to change their habits [4, 5]. Recent studies suggest that dietary supplementation of certain nature products, such as fish oil-derived Omega-3 polyunsaturated fatty acids (n-3 PUFA) [6–10] and/or plant derived-PUFA, phospholipids, sterols and stanols can be beneficial on blood cholesterol and blood pressure [11–26]. However, controversy still remains around the cardioprotective effects of fish oil-derived n3-PUFA [27–29] probably due to heterogeneous nature in the selection of different participants, formulation of different supplements, background diet, ratio of n3-PUFA: n6-PUFA and outcome measures [30–32].

The aim of this observational study was to evaluate the effects of supplementation of Omega3Q10, a fish oil-based Omega-3 n-3 PUFA formulation and soybean oil control in older adults with elevated blood pressure and blood cholesterol.

## Methods

### Study population

The recruitment of the participants into the study was started on September 2, 2013 and completed on October 12, 2013, and the last follow-up observation was completed on January 7, 2014, at Tang-Qiao Community Health Service Center in Pudong New District in Shanghai, China. Volunteers were recruited by self-referral in response to media coverage and word of mouth. All study procedures were conducted in accordance with the Helsinki Declaration of 1975 and were approved by the Shanghai Jiao Tong University School of Public Health Institutional Review Board. Written informed consent form was obtained from all participants prior to enrollment into the study.

Subjects who met the first and one of other three following criteria were eligible for the study:

**Inclusion criteria:**

1. Healthy males or females at least 50 years of age;
2. Having current symptoms of elevated blood pressure (systolic blood pressure 130–139 mmHg or diastolic blood pressure 85-89 mmHg);
3. Having a history of elevated blood pressure (systolic blood pressure 140-159 mmHg or diastolic blood pressure 90-99 mmHg);
4. Having a history of elevated blood cholesterol (total cholesterol (TC) concentrations higher than normal range: 2.8 to 5.7 mmol/L; HDL-C: 0.78 to 1.55 mmol/L; LDL-C: 1.68 to 4.53 mmol/L; glucose: 3.9 to 6.1 mmol/L).

**Exclusion criteria:**

1. Having diagnosed with any severe medical conditions or complications of the liver, kidneys, heart, lungs, or any other organs, or malignant tumors;
2. People who have much doubts of the study, unwilling to participate or unlikely to keep adherence of the study procedure were also excluded.

### Randomization and blindness

Participants were randomly assigned to the Omega3Q10 treatment group or soybean oil control group. The randomization was performed using a predetermined randomization code which was generated by a random number generator.

Trial participants and community doctors were both blinded from the treatment (double-blind trial). Of the 100 enrolled participants, 97 participants completed the 12-week follow-up, including 48 subjects in the Omega3Q10 group and 49 subjects in the control group. Two subjects of the Omega3Q10 group and 1 subject of the soybean oil control group withdrew from the study due to objections by family members.

The participants received similar-looking capsules in color-coded bottles (white bottles for Omega3Q10 and yellow bottles for soybean oil control). Neither the subjects nor the medical doctors, including the study principal investigator (R.S.), knew the specific color code until the end of the study. Both the Omega3Q10 capsules and the control capsules (which was composed of soybean oil) were manufactured and supplied by GardaVita® Inc. (Costa Mesa, California, USA). Each participant was instructed to take 1 capsule with a meal, two times per day for 12 weeks and a new batch of supplements was dispensed every month during follow-up sessions.

The key active ingredients of Omega3Q10 formulation include Omega-3 fish oil (1000 mg/day) (including EPA (eicosapentaenoic acid, 312 mg), DHA (docosahexaeonic acid, 202 mg), Omega3Q10 complex (L-carnitine fumarate, Co-enzyme Q10, lycopene, 265 mg), vitamin E (alpha tocopherol acetate and natural tocopherol oil, 50 IU), Vitamin B6 (pyridoxine hydrochloride, 20 mg), B12 (methylcobalamin, 500 mcg) and folic acid (400 mcg). Each soybean oil capsule includes 910 mg soybean oil (saturated fatty acids 31.3 mg); monounsaturated fatty acids (45.57 mg); polyunsaturated fatty acids (229.48 mg); oleic acid (48 mg).

### Evaluation of blood pressure, blood lipids/ cholesterols and quality of life

Blood concentrations of TC, LDL-C, HDL-C were evaluated before and after the 12-week treatment. Changes in blood pressure and in elevated blood pressure-related symptoms including headache, dizziness, tinnitus, palpitations, and chest tightness were evaluated before and after the 12-week intervention. Measurement of baseline blood pressure, and the measurement of the final blood pressure were both repeated twice with each repeat separated by a 1-week time interval. Elevated blood pressure-associated symptoms i.e. dizziness, headache, tinnitus, palpitations and chest tightness were scored using a self-administered 6-point scale (0 = no symptoms, 5 = most severe). All participants were followed up each month in order to check compliance and adverse effects.

### Statistics analysis

EpiData 3.1 software was used for the data entry and SPSS 20 software was used for statistical analysis. Group data were presented as the mean ± standard deviation or median ± Quartile Range (QR). Differences between the Omega3Q10 and soybean oil groups were compared using Student’s t-test for quantitative variables with normal distribution and Mann-Whitney U test for variables with non-normal distribution, or Chi-square test for categorized variables. Ridit scoring test, which is a non-parameter test for comparing two or more sets of ordered qualitative data, was used for evaluating the changes in the symptom severity scores after the intervention. A multivariate regression analysis was also performed in order to evaluate the role of confounding factors on final results. The alpha level of *P* > 0.05 was chosen as being statistically significant. All *p*-values reported were 2-sided.

## Results

### Demographic characteristics

The baseline information of age, gender and histories of alcohol intake, disease and medication of the participants are shown in Table 1. There were 24 males (50%) and 24 females (50%) in the Omega3Q10 group, and 24 males (48.98%) and 25 females (51.02%) in the soybean oil group. The gender distribution between the two groups was not significantly different between the two groups (χ^2^ = 0.10, *P* = 0.920). The average age of all participants was 62.83 ± 10.16, and no significant difference for age was found between the Omega3Q10 group (63.26 ± 10.34 years) and the soybean oil group (62.41 ± 10.10 years) (*t* = 0.411, *P* = 0.682).

**Table 1:** Demographics and medical history of the participants

	Group	Male	Female	Sub Total
Gender, N (% of subtotal)	Omega3Q10	24 (50.0%)	24 (50.0%)	48 (100%)
	Soybean oil	24 (48.98%)	25 (51.02%)	49 (100%)
	Combined	48 (49.48%)	49 (50.52)	97 (100%)
Age, year (mean ± s.d)	Omega3Q10	63.87 ± 10.33	62.66 ± 10.54	63.26 ± 10.34
	Soybean oil	60.67 ± 9.47	64.07 ± 10.60	62.41 ± 10.10
	Combined	62.27 ± 9.93	63.38 ± 10.48	62.83 ± 10.16
Age, year (range)	Omega3Q10	50.06–83.30	45.39–84.32	45.39–84.32
	Soybean oil	48.90–77.91	49.81–83.79	48.90–83.79
	Combined	48.90–83.30	45.39–84.32	45.39–84.32
Smoking, Yes/total (%)	Omega3Q10	11/24 (45.83)	0	11/48 (22.92)
	Soybean oil	10/24 (41.67)	0	10/49 (20.41)
	Combined	21/48 (43.75)	0	23/97 (21.65)
Alcohol drinking, Yes/total (%)	Omega3Q10	11/24 (45.83)	0	11/48 (22.92)
	Soybean oil	12/24 (50.0)	0	12/49 (24.49)
	Combined	23/48 (47.92)	0	23/97 (23.71)
Hypercholesterolemia, Y/T, (%)	Omega3Q10	12/24 (50.0)	10/24 (41.67)	22/48 (45.83)
	Soybean oil	9/24(37.5)	7/25(28.0)	16/49 (32.65)
	Combined	21/48 (43.75)	17/49 (34.69)	38/97 (39.18)
Elevated Blood Pressure, Y/T, (%)	Omega3Q10	12/24 (50.0)	7/24 (29.17)	19/48 (39.58)
	Soybean oil	11/24(45.83)	12/25(48.0)	23/49 46.93)
	Combined	23/48 (47.92)	19/49 (38.78)	42/97 (43.30)
Coronary Heart Issues, Y/T, (%)	Omega3Q10	1/24 (4.17)	2/24 (8.33)	3/48 (6.25)
	Soybean oil	3/24 (12.5)	5/25 (20.0)	8/49 (16.33)
	Combined	4/48 (8.33)	7/49 (14.29)	11/97 (11.34)
Stroke, Yes/total (%)	Omega3Q10	0	1/24 (4.17)	1/48 (2.08)
	Soybean oil	1/24 (4.17)	2/25 (8.0)	3/49 (6.12)
	Combined	1/48 (2.08)	3/49 (6.12)	4/97 (4.12)
High Blood Sugar, Yes/total (%)	Omega3Q10	2/24 (8.33)	1/24 (4.17)	3/48 (6.25)
	Soybean oil	1/24 (4.17)	3/25 (12.0)	4/49 (8.16)
	Combined	3/48 (6.25)	4/49 (8.16)	7/97 (7.21)
Kidney issues, Yes/total (%)	Omega3Q10	0	0	0
	Soybean oil	0	0	0
	Combined	0	0	0

There were no significant differences between Omega3Q10 and soybean oil groups in the patterns of alcohol drinking (22.9% vs. 24.5%) (χ^2^ = 0.033, *P* = 0.855), smoking (22.9% vs. 20.4%) (χ^2^ = 0.09, *P* = 0.764), medical history of elevated blood pressure (39.6% vs. 46.9%) (χ^2^ = 0.534, *P* = 0.465), hypercholesterolemia (45.8% vs. 32.7%) (χ^2^ = 1.768, *P* = 0.184), coronary heart issues (6.2% vs. 16.3%) (χ^2^ = 2.449, *P* = 0.118), stroke (2.1% vs. 6.1%) (χ^2^ = 0.24, *P* = 0.624), diabetes (6.2% vs. 8.2%) (χ^2^ = 0.0, *P* = 1.00), and other health-related concerns (2.1% vs. 2.0%) (χ^2^ = 0.0, P = 1)(Table 1).

There were no significant differences between Omega3Q10 and soybean oil groups in the proportion of the participants who used anti-hypertensive medicine (35.4% vs. 46.9%,*χ* ^2^=1.328,*P* = 0.249, Renin-angiotensin system inhibitors (ACEI/ARB): 20.83% vs. 20.41%; calcium channel blockers: 10.42% vs. 16.33%; Diuretics: 2.08% vs. 4.8%; β-blockers: 2.04% vs. 1.03%% and other anti-hypertensive medicine: 2.04% vs. 4.08%), the number of years of anti-hypertensive medicine use (5.7 ± 4.0 vs. 5.8 ± 6.7,Z = 0.793,*P* = 0.428), the proportion of the participants who used cholesterol lowering drugs (6.7% vs. 11.6%, *χ* ^2^=0.192,*P* = 0.661) and the year of cholesterol lowering drug use (1.4 ± 1.4 vs. 2.5 ± 4.2,*t* = 0.426, *P* = 0.685).

### Blood lipids/cholesterol levels

There are no significant differences between Omega3Q10 and soybean oil groups in baseline blood cholesterol (5.5 ± 0.7 mmol/L vs. 5.7 ± 0.8 mmol/L) (*P* > 0.05), HDL (1.3 ± 0.6 mmol/L vs. 1.0 ± 0.8 mmol/L) (*P* > 0.05), and LDL (3.7 ± 0.8 mmol/L vs. 3.6 ± 0.7 mmol/L), (*P* > 0.05), nor in post-treatment blood cholesterol (5.3 ± 0.5 mmol/L vs. 5.6 ± 0.7 mmol/L) (*P* > 0.05), HDL (1.3 ± 0.5 mmol/L vs. 1.2 ± 0.7 mmol/L) (*P* > 0.05), and LDL (3.3 ± 0.6 mmol/L vs. 3.4 ± 0.8 mmol/L) (*P* > 0.05) (Table 2). Both Omega3Q10 and soybean oil treatments, however, significantly reduced cholesterol and LDL-C and increased HDL-C (*P* < 0.05, each), with a relatively greater reduction of TC and LDL-C found in the Omega3Q10 group than in soybean oil group.

**Table 2:** Blood concentrations of cholesterol, LDL-C, HDL-C and TC before and after 12-weeks intervention in Omega3Q10 and soybean oil groups

Blood metabolites		Before Intervention	After Intervention
		mmol/L (Median ± QR)	mmol/L (Median ± QR)
LDL-C	Omega3Q10	3.7 ± 0.8	3.3 ± 0.6*
	Soybean oil	3.6 ± 0.7	3.4 ± 0.8*
	(Z, *P*)	0.65, 0.51	0.57, 0.57
HDL-C	Omega3Q10	1.25 ± 0.57	1.31 ± 0.54*
	Soybean oil	1.0 ± 0.8	1.2 ± 0.7*
	(Z, P)	0.90, 0.37	0.50, 0.62
TC	Omega3Q10	5.5 ± 0.7	5.3 ± 0.5*
	Soybean oil	5.7 ± 0.8	5.6 ± 0.7*
	(Z, P)	0.38, 0.70	0.71, 0.48

*p < 0.05, within-group comparison of the after-treatment value with the baseline

### Blood pressure

The mean values of systolic blood pressure (SBP) and diastolic blood pressure (DBP) of the Omega3Q10 group and the soybean oil group before and after the 12-week treatment are shown in Table 3. There were no baseline differences between the Omega3Q10 and soybean oil groups in SBP (132.3 ± 5.5 vs.131.8 ± 9.3, *P* > 0.05) and DBP (81.6 ± 5.3 vs. 80.4 ± 6.1, *P* > 0.05) (Table 3), nor after the 12-week treatment in SBP (130.7 ± 7.3 vs. 129.7 ± 8.7 *P* > 0.05) and DBP (79.3 ± 5.2 vs. 79.1 ± 5.3, *P* > 0.05, respectively) (Tables 3). However, both SBP and DBP levels were reduced after either Omega3Q10 or soybean oil treatment, with a significant DBP reduction only found in the Omega3Q10 group. There are no baseline differences in the blood pressure equilibrium distribution (normal, high-normal (blood pressure), or level I hypertension (mild)) according to the Guidance for the prevention and management of elevated blood pressure (2010 Chinese Edition) between the two groups (*χ* ^2^=0.907, *P* = 0.341) (Table 4).

**Table 3:** Mean blood pressures (mean ± s.d) before and after the 12-week intervention in Omega3Q10 and soybean oil groups

Blood pressure (BP, mmHg)	Before intervention	After intervention	Declined value
Systolic BP			
Omega3Q10	132.3 ± 5.5	130.7 ± 7.3	1.6 ± 7.3
Soybean oil	131.8 ± 9.3	129.7 ± 8.7	2.0 ± 10.4
(t, *P*)	0.36, 0.72	0.58, 0.56	0.22, 0.83
Diastolic BP			
Omega3Q10	81.6 ± 5.3	79.3 ± 5.2*	2.3 ± 5.9
Soybean oil	80.4 ± 6.1	79.1 ± 5.3	1.3 ± 7.9
(t, *P*)	1.05, 0.30	0.20, 0.84	0.71, 0.48

*p < 0.05, within-group comparison of the after-treatment value with the baseline

**Table 4:** Distribution of people with normal BP, pre-hypertension and hypertension in Omega3Q10 and soybean oil groups

Blood pressure	Omega3Q10		Soybean oil		Total	
	n	%	n	%	n	%
Baseline						
Normal	0	0.0	4	8.2	4	4.1
Pre-hypertension	40	83.3	33	67.3	73	75.3
Hypertension	8	16.7	12	24.5	20	20.6
After intervention						
Normal	1	2.1	5	10.2	6	6.2
Pre-hypertension	35	72.9	31	63.3	66	68.0
Hypertension	12	25.0	13	26.5	25	25.8

### Hypertension-related symptoms

The results of Mann-Whitney U test showed no baseline differences between the Omega3Q10 and soybean oil groups in the self-reported hypertension-related symptom severity scores of headaches (Z = 1.585, *P* = 0.113), tinnitus (Z = 0.581, *P* = 0.561) and palpitations & chest tightness (Z = 0.92, *P* = 0.358) except a significant group difference in the distribution of dizziness scores (Z = 2.785, *P* = 0.005) (Table 5). After the 12-week intervention, however, more people in the Omega3Q10 group showed reduced symptom severities of headache (Z = 3.238, *P* = 0.001); tinnitus (Z = 1.732, *P* = 0.083) and; palpitations & chest tightness (Z = 2.852, *P* = 0.004) than the soybean oil group (Tables 6).

**Table 5:** Baseline differences in blood pressure-related symptom severity scores between Omega3Q10 and soybean oil groups (0 = no symptoms, 5 = most severe)

Symptom	Group	Symptom score (N/%)						Z	P
		0	1	2	3	4	5		
Dizziness	Omega3Q10	31/64.6	6/12.5	6/12.5	1/2.1	4/8.3	0/0.0	2.785	0.005
	Soybean oil	16/32.7	13/26.5	11/22.4	3/6.1	6/12.2	0/0.0		
Headache	Omega3Q10	36/75.0	5/10.4	4/8.3	2/4.2	1/2.1	0/0.0	1.585	0.113
	Soybean oil	28/57.1	13/26.5	5/12.2	1/2.0	2/4.1	0/0.0		
Tinitus	Omega3Q10	38/79.2	6/12.5	3/6.3	1/2.1	0/0.0	0/0.0	0.581	0.561
	Soybean oil	37/75.5	5/10.2	3/6.1	3/6.1	1/2.0	0/0.0		
Palpitations & chest tightness	Omega3Q10	40/83.3	3/6.3	2/4.2	2/4.2	1/2.1	0/0.0	0.920	0.358
	Soybean oil	37/75.5	3/6.1	7/14.3	1/2.0	1/2.0	0/0.0		
Other symptoms	Omega3Q10	47/97.9	1/2.1	0/0.0	0/0.0	0/0.0	0/0.0	1.016	0.310
	Soybean oil	46/93.9	1/2.0	1/2.0	1/2.0	0/0.0	0/0.0

**Table 6:** Differences in hypertension-related symptom severity scores between Omega3Q10 and soybean oil groups after the 12-weeks intervention (0 = no symptoms, 5 = most severe)

Symptom	Group	Symptom score difference (N/%)						Z	P
		0	1	2	3	4	5		
Dizziness	Omega3Q10	28/58.3	16/33.3	4/8.3	0/0.0	0/0.0	0/0.0	0.795	0.427
	Soybean oil	27/55.1	12/24.5	8/16.3	2/4.1	0/0.0	0/0.0		
Headache	Omega3Q10	46/95.8	2/4.2	0/0.0	0/0.0	0/0.0	0/0.0	3.238	0.001
	Soybean oil	35/71.4	12/24.5	2/4.1	0/0.0	0/0.0	0/0.0		
Tinitus	Omega3Q10	48/100.0	0/0.0	0/0.0	0/0.0	0/0.0	0/0.0	1.732	0.083
	Soybean oil	46/93.9	1/2.0	1/2.1	0/0.0	1/2.0	0/0.0		
Palpitations & chest tightness	Omega3Q10	46/95.8	2/4.2	0/0.0	0/0.0	0/0.0	0/0.0	2.852	0.004
	Soybean oil	37/75.5	10/20.4	1/2.1	1/2.0	1/2.0	0/0.0		
Other symptoms	Omega3Q10	48/100.0	0/0.0	0/0.0	0/0.0	0/0.0	0/0.0	<0.001	1.000
	Soybean oil	49/100.0	0/0.0	0/0.0	0/0.0	0/0.0	0/0.0		

## Discussion

Elevated blood lipid level and high-than-normal blood pressures are independent risk factors of increased cardiovascular dysfunction, chronic inflammation and stroke [33–36]. Despite the development of pharmaceutical therapies and the recommendations of life style modification, many people still fail to achieve therapeutic goals [37–39]. Dietary supplements of fish oil-derived n-3 PUFA or fish oil n-3 PUFA may reduce plasma cholesterol and LDL-C levels, and cardiovascular risk [18, 20].

In this study, both Omega3Q10 and soybean oil significantly reduced blood cholesterol and LDL-C levels and, to a less extend, reduced blood pressures by Omega3Q10. in old adults with elevated blood pressure and blood cholesterol levels. Omega3Q10 also reduced the symptom severities of headache and palpitations & chest tightness. These results are in agreement with the current knowledge that intake of n-3 polyunsaturated fatty acid (PUFA) especially that of fish oil can reduce the incidence of hypertension and cardiac mortality in certain subpopulation [40–42], and in line with the recent report that 12 months of omega 3 PUFA supplementation significantly decreased systolic blood pressure (SBP) (by 2.7 +/− 2.5 mmHg, *p* = 0.001) and diastolic blood pressure (DBP) (by 1.3 +/− 3.3 mmHg, *p* < 0.001), in 111 hypertriglyceridemic patients with untreated normal-high blood pressure and with or without metabolic syndrome [43].

Soybean oil was also effective in improving dyslipidemia probably because soybean oil contains n3-PUFA or other potent ingredients that have cholesterol relieving activities. Sterols, sterol esters and the sterol glycosides present in soybean oil [44], can inhibit cholesterol absorption and lower LDL-C levels [45].

In this study, the daily dose of Omega3Q10 contains 514 mg PUFA (312 mg EPA (n3-PUFA) and 202 mg DHA (n6-PUFA) whereas the daily dose of soybean oil contains 115.48 mg PUFA (14 mg linolenic acid (n3-PUFA), 100 mg linoleic acid (n6-PUFA) and 48 mg oleic acid all of which have different cholesterol and LDL-C lowering effects as well as cardioprotective effects albeit with the fish n-3 PUFA having better efficacy. Other studies showed that a 45-day supplement of high dose of fish oil-derived n-3 PUFA (3 g/day) significantly reduced systolic pressure [17]; high blood n-3 fatty acids levels are associated with reduced risk of death, [46] and dietary intake of linolenic acid is associated with a lower risk of cardiovascular disease mortality. [47]. Additionally, eating fish monthly can reduce the risk of ischemic stroke. [48]. Epidemiological studies also support a protective role of plant-derived n-3 fatty acid such as alpha-linolenic acid (ALA), which was inversely related to all-cause mortality in 5,452 participants without prior cardiovascular disease [49].

Genetic background may affect the efficacy of fish oil-derived n-3 PUFA. A recent longitudinal study shows that a higher omega-3 PUFA intake was associated with a more pronounced blood pressure decrease over time in subjects with the CYP4F2 433VV genotype [50]. Omega-3 PUFA may also reduce blood pressure by acting as a substrate of cytochrome P450 (CYP450), the enzymes involved in the production of vasoactive mediators. Functional polymorphisms (SNPs) in CYP450 genes are associated with elevated blood pressure and ischemic stroke [51–56]. Dietary fish oils have been shown to have different preventive effects on the development of elevated blood pressure and vascular response in different genetic strains of rat [57]. Depending on rat strains and fish oil composition, this was more pronounced for fish oils enriched with EPA and DHA and was more prominent in the SHR and SHR/WKY backcross than it was in the SHR-SP.

There is a possibility that ingredients such as vitamin B of the Omega3Q10 formulation may have interacted with n3- PUFA and n6-PUFA on cholesterol and LDL-C levels as supplementation of B vitamins had no beneficial effects on health-related quality of life in stroke and cardiovascular disease survivors [58].

Fish oil-derived n-3 PUFA may exert its cardioprotective effects by anti-inflammatory mechanisms [59]. Long-term n3-PUFA dietary supplementation prevents the development of intracranial atherosclerosis and macrophage infiltration into the vessel wall, therefore reducing inflammation and initial thickening in animals [60]. Omega-3 PUFAs may exert their multiple health-benefit effects partly by actions such as antagonizing arachidonic acid-induced proinflammatory prostaglandin E_2_ (PGE_2_) formation and by suppressing nuclear factor-κB, C-reactive proteins that are potent inducer of proinflammatory cytokines including interleukin-1beta, interleukin 6 and tumor necrosis factor-α, which are decreased by EPA and DHA [61–67] [68]. n-3 PUFAs can also repress lipogenesis and increase resolvins and protectin generation, thus reduced inflammation. EPA and DHA benefit insulin resistance by inducing adiponectin, an anti-inflammatory adipokine [67].

One study, however, showed that treatment with statin alone or statin plus fish oil, but not fish oil alone, reversed the increased plasma hs-CRP and IL-6 (a low-grade chronic inflammatory state) in in 48 obese individuals [69]. Marine omega-3 fatty acids also dose-dependently regulated apolipoproteins, apolipoprotein-defined lipoprotein subclasses, and Lp-PLA2 in individuals with moderate hypertriglyceridemia [70]. It is known that apolipoprotein (apo) distribution and lipoprotein (Lp)-associated markers of inflammation, such as lipoprotein-associated phospholipase A2 (Lp-PLA2), influence the atherogenicity of circulating lipids and lipoproteins.

Other multiple mechanisms may underlie the beneficial effect of Omega3O10. It has been reported that the antihypertensive effect of angiotensin converting enzyme (ACE) inhibitors can be augmented by supplementing fish oil [71]. Omega-3 PUFAs could regulate vasomotor tone and renal sodium excretion, through competing with omega-6 PUFAs for common metabolic enzymes and thereby decreasing the production of vasoconstrictor. PUFAs also reduce angiotensin-converting enzyme (ACE) activity, angiotensin II formation, tumor growth factor-beta (TGF-b) expression, enhance endothelial nitric oxide (NO) generation and activate the parasympathetic nervous system, resulting in improved vasodilation and arterial compliance of both small and large arteries [72]. Indeed, fish oil attenuated adrenergic overactivity without altering glucose metabolism during an oral glucose load [73]. Omega-3 fatty acids improved platelet redox balance in diabetic patients with hypertension [74]. Fish oil supplements increased the concentration of phospholipid, improved membrane fluidity, decreased the activity of HMG-CoA reductase in alloxan-induced diabetic mice [75]. n-3 PUFA is also involved in the regulation of mitochondrial 3-hydroxy-3-methylglutaryl-CoA (HMG-CoA) synthase (HMGCS2) that catalyzes the first step of ketogenesis and is critical in various metabolic conditions [76].

In this study, participants who received soybean oil capsule also showed improved cholesterol and LDL-C levels. Studies have shown that, soybean oil contains n-3 PUFA and other active ingredients such as proteins, phospholipids, lecithin, sterol and stanol, all of which can affect cholesterol metabolism. Supplement of soybean-derived lecithin for 2 years reduced serum cholesterol and triglycerides levels by 22% and 26%, respectively, in people on low fat diet. [77]. Replacement of dietary animal proteins with soy proteins for 4 weeks significantly reduced blood cholesterol by 18.6% and improved HDL-C, in 65 people with hyperlipidemia [78]. And novel soybean oils differing in linoleic acid (LA) and alpha-linolenic acid (ALA) ratio and n-6/n-3 ratio can alter immune functions. [79], and serum cholesterol and triglyceride levels, respectively [80].

Several meta-analyses reported that daily intake of up to 2 g of stanols or sterols can reduce blood LDL-C by 10% (or by 20% if on low fat/cholesterol diet or on statin medication) [81–84]. However, because sterol-enriched foods increase plasma sterol levels and the risk of atherosclerosis in patients with homozygous phytosterolemia [45, 85, 86], further studies are needed to determine the optimal dose range in different subpopulations.

### Limitations

There are limitations of this pilot study. The number of the participants, the selection of the dosage of Omega3Q10 supplement and the control group (soy oil) were not optimized for detecting potential effects of Omega3Q10 on the studied parameters. For example, the originally calculated sample size (*N* = 45 per group, with 10% drop rate) was based on the assumption of a baseline value of 3.7 ± 0.7 mmol/L of LDL-C in the Omega3Q10 and soybean oil group that would be decreased to 3.3.0 ± 0.6 mmol/L after 12-week Omega3Q10 intervention and no change after soybean oil intervention. But the results show that soy oil tended to produce similar effects as that of Omega3Q10 probably due to the presence of plant sterols and other active PUFA ingredients in soy oil.

The concurrent use of different anti-hypertension and anti-dyslipidemia drugs by a significant portion of the participants in the Omega3Q10 group (42.1%) and in the Soy Oil group (58.5%) would also make it difficult to discern the true effects of the Omega3Q10 and Soy oil on blood pressure and blood lipid profile.

Previous studies showed that both statin therapy (simvastatin (40 mg/d) and co-supplements of red yeast rice (a natural source of statins) and fish oil for 12 weeks significantly reduced LDL-C levels (−39.6% vs. −42.4%, *P* < .001 and *P* < .001) [87]. And co-administration of omega-3 fatty acids (4.2 g/d) with statin for 8-weeks significantly increased LDL particle size and decreased TG level in dyslipidemic patients with type 2 diabetes and without causing significant adverse effects [88]. Furthermore, co-administration of omega-3 (EPA 465 mg; DHA, 375 mg; othern-3 FA, 60 mg) and statins (simvastatin 20 mg /d) for 6 weeks produced greater reduction in TG than statin monotherapy (41% reduction vs 13.9% reduction, *P* < 0.01) [89].

Other studies show that statin alone or co-treatment with fish oil, but not fish oil alone, reversed increased plasma hs-CRP and IL-6 and, hence reduced chronic inflammatory state in visceral obesity [69]. In addition, the triglyceride-lowering effect of atorvastatin, but not fish oils, is associated with increased VLDL apo C-III fractional catabolism and hence lower VLDL apo C-III concentrations. Co-treatment provided no significant additional improvement in VLDL apo C-III metabolism compared with atorvastatin alone [90].

Recent studies have also showed that 16-week of co-administration of plant sterols esters (1300 mg), fish oil (1000 mg EPA and DHA) and vitamins B12 (50 mug), B6 (2.5 mg), folic acid (800 mug) and coenzyme Q10 (3 mg) significantly reduced total cholesterol, LDL- cholesterol, VLDL-cholesterol, subfractions LDL-2, IDL-1, IDL-2 and plasma homocysteine levels and decreased triacylglycerols levels at a trend level (by 17.6%) in hypercholesterolemic children and adolescents [91]. Fish-oil esters of plant sterols improve the lipid profile of dyslipidemic subjects more than do fish-oil or sunflower oil esters of plant sterols [92]. Low doses of fish oil-derived EPA and DHA plus plant sterols also dose-dependently decreased serum triglyceride concentrations in hypercholesterolemic men and women [93]. These studies together with our current findings suggest potential additive benefits of co-administration of n-3 PUFAs and plant sterols for people with increased risk of cardiovascular conditions and that should be evaluated in future better-controlled, large-scale studies.

In this study, we also conducted multivariate regression analysis, with intervention groups as the primary variable of interest. The covariates were baseline smoking status (yes/no), current medicine use (yes/no), baseline DBP (mmHg), and baseline cholesterol level (baseline TC/baseline HDL/baseline LDL). No significant differences were found between the Omega3Q10 and soybean oil in blood pressures (SBP/DBP) or in cholesterol (LDL/TC/HDL) after the potential confounders were controlled, suggesting that both Omega3Q10 and soybean oil supplementation had similar effects on blood cholesterol and blood pressure. We did notice, however, that after controlling the current medicine use (taken by 42.1% of the Omega3Q10 group and by 58.5% of the Soy Oil group), the LDL-C results showed a trend difference (*P* < 0.18) between the Omega3Q10 and Soybean oil group. Because the number of people is very small and the medicine varied greatly (anti-hypertension or lipid-lowering etc.), further studies should determine if Omega3Q10 and soybean oil could interact differently with different anti-hypertension and anti-dyslipidemia drugs.

## Conclusions

Dietary supplementation of Fish oil-based Omega3Q10 were safe and effective in reducing blood pressure and blood cholesterol and LDL-C levels in old adults with hypertension and hypercholesterolemia. Omega3Q1 also appears to be more effective than soybean oil in improving DBP and hypertension-related symptoms in old adults with hypertension and hypercholesterolemia although both supplementation improved TC, LDL-C and HDL-C concentrations.

## Abbreviations

ALA: alpha-linolenic acid
CYP450: cytochrome P450
DBP: diastolic blood pressure
DHA: docosahexaeonic acid,
EPA: eicosapentaenoic acid
HDL-C: high density lipoprotein cholesterol
LA: linoleic acid
LDL-C: low density lipoprotein cholesterol
n3-PUFA: omega-3 poly-unsaturated fatty acids
Omega3Q10: dietary supplement formulation based on marine n3-PUFA
SBP: systolic blood pressure
SHR: spontaneously hypertensive rat
SHR/WKY: a backcross of SHR and WKY rats
SHR-SP: stroke-prone spontaneously blood pressure symptoms rats
SNPs: single nucleotide polymorphisms
TC: total cholesterol
WKY: Wistar-Kyoto rat, a normotensive and the closest genetic control for the SHR rats
